# Supplementary material for: Thermochemical and Toxic Element Behavior during Co-Combustion of Coal and Municipal Sludge
Source: Molecules. 2021 Jul 9;26(14):4170. doi: 10.3390/molecules26144170 (PMC8307717; doi:10.3390/molecules26144170)
Supplement: Supplementary file 1 [file molecules-26-04170-s001.zip › molecules-1239433-supplementary.pdf]

Table S1 Combustion characteristics for coal, municipal sludge and their blends at heating rate of 10 (a), 20 (b) and 60 (c) °C/min.

(a)

Sample	Stage II				Stage III			
	Range (°C)	T_{max} (°C)	DTG_{max} (%/°C)	W (%)	Range (°C)	T_{max} (°C)	DTG_{max} (%/°C)	W (%)
C					301-601	505	1.576	56.5
C90MS10	209-299	270	0.019	0.28	299-609	505	1.315	53.4
C80MS20	199-304	284	0.082	1.50	304-612	508	1.302	50.8
C70MS30	194-325	281	0.147	3.02	325-632	508	1.170	46.9
C60MS40	189-341	284	0.221	6.25	341-640	508	1.148	41.8
C50MS50	182-353	285	0.257	8.75	353-601	515	0.884	36.5
MS	187-368	282	0.596	19.7	369-620	282	0.596	13.7

(b)

Sample	Stage II				Stage III			
	Range(°C)	T_{max}(°C)	DTG_{max}(%/°C)	W (%)	Range (°C)	T_{max} (°C)	DTG_{max} (%/°C)	W (%)
C					314-651	529	2.478	56.2
C90MS10	188-319	287	0.063	0.80	319-635	530	2.587	53.9
C80MS20	200-320	296	0.162	1.79	320-689	528	2.368	50.9
C70MS30	202-344	298	0.286	3.97	344-651	537	1.853	46.5
C60MS40	203-355	293	0.393	6.32	355-646	539	1.555	41.4
C50MS50	200-364	295	0.543	8.39	364-654	542	1.578	37.4
MS	201-380	293	1.050	19.1	380-659	430	0.649	14.9

(c)

Sample	Stage II				Stage III			
	Range (°C)	T_{max} (°C)	DTG_{max} (%/°C)	W (%)	Range (°C)	T_{max} (°C)	DTG_{max} (%/°C)	W (%）
C					337-763	578	3.894	56.9
C90MS10					253-838	615	2.401	54.2
C80MS20	226-356	333	0.401	2.38	356-831	606	2.174	49.8
C70MS30	225-366	331	0.678	4.20	366-731	600	2.930	45.5
C60MS40	219-386	326	0.987	6.80	386-759	603	2.474	41.3
C50MS50	222-394	324	1.346	9.05	394-793	610	2.016	36.7
MS	225-406	317	2.613	18.5	406-673	469	1.744	15.2

Notes: T_{max}, the temperature at which the mass loss rate is maximum; DTG_{max}, the maximum rate of weight loss; W, the weight loss in an individual stage.
